# Mechanisms of Melatonin in Obesity: A Review

**DOI:** 10.3390/ijms23010218

**Published:** 2021-12-25

**Authors:** Qingyun Guan, Zixu Wang, Jing Cao, Yulan Dong, Yaoxing Chen

**Affiliations:** Neurobiology Laboratory, College of Veterinary Medicine, China Agricultural University, Haidian, Beijing 100193, China; qyguan@cau.edu.cn (Q.G.); zxwang@cau.edu.cn (Z.W.); caojing@cau.edu.cn (J.C.); ylbcdong@cau.edu.cn (Y.D.)

**Keywords:** melatonin, obesity, lipid metabolism, mechanisms

## Abstract

Obesity and its complications have become a prominent global public health problem that severely threatens human health. Melatonin, originally known as an effective antioxidant, is an endogenous hormone found throughout the body that serves various physiological functions. In recent decades, increasing attention has been paid to its unique function in regulating energy metabolism, especially in glucose and lipid metabolism. Accumulating evidence has established the relationship between melatonin and obesity; nevertheless, not all preclinical and clinical evidence indicates the anti-obesity effect of melatonin, which makes it remain to conclude the clinical effect of melatonin in the fight against obesity. In this review, we have summarized the current knowledge of melatonin in regulating obesity-related symptoms, with emphasis on its underlying mechanisms. The role of melatonin in regulating the lipid profile, adipose tissue, oxidative stress, and inflammation, as well as the interactions of melatonin with the circadian rhythm, gut microbiota, sleep disorder, as well as the α7nAChR, the opioidergic system, and exosomes, make melatonin a promising agent to open new avenues in the intervention of obesity.

## 1. Introduction

There is no doubt that obesity has become a challenging global public health crisis. Previously, it was determined that people with overweight or obesity were over 2 billion, which comprised one-third of the worldwide population [1]. Obesity can lead to many other dysfunctions, such as type 2 diabetes mellitus (T2DM), dyslipidemia, nonalcoholic fatty liver disease (NAFLD), cardiovascular disease [2], making obesity and its complications a more pivotal issue. Thus, promising strategies are urgently needed to impede the progression of obesity.

Melatonin, which is found in nearly all organisms from primitive photosynthetic bacteria to humans [3], is an endogenous indoleamine hormone that participates in various physiological processes. Controlled by the hypothalamic suprachiasmatic nucleus (SCN), melatonin is rhythmically synthesized in the pineal gland in vertebrates [4], and exerts powerful physiological functions via melatonin receptor 1 (MT1) and melatonin receptor 2 (MT2) [5], G-protein-coupled membrane receptors, in mammals. Currently, melatonin is understood as a pleiotropic hormone that plays outstanding effects on the circadian rhythm [6], immune system [7], cancers [8], and even energy metabolism [9]. During the past decade, the link between melatonin and glucose metabolism as well as T2DM has been established despite some controversial outcomes. Given that melatonin is a potential regulator of metabolism, the relationship between melatonin and obesity has been discussed in some existing literature reviews [10,11,12], involving the role of melatonin in regulating obesity and the recognized mechanisms, such as antioxidant and anti-inflammatory effects. In this review, we mainly focus on the mechanisms of melatonin in obesity, especially the potential pathways that have not been summarized yet, in a more systematic and in-depth manner, thus providing more insight for obesity study and prevention.

## 2. Effect of Melatonin on Obesity

### 2.1. Body Weight

As early as 1984, Bartness et al. found that the short photoperiod induced weight gain in hamsters after pinealectomy [13], which suggested that there was a relationship between the pineal gland, melatonin, and body weight. Growing evidence subsequently did show that the exogenous melatonin supplementation reduced body weight in animals [14]. Melatonin inhibited weight gain and related phenotypes such as visceral fat deposition in many animal models, especially in those fed a high-fat/high-sugar diet (Table 1).

In clinical, previous evidence that shows the weight loss effect of melatonin was relatively weak. Besides, some of the investigations of the association between melatonin and body weight focus on the role of melatonin in some clinical drugs used for patients with mental problems, which can inevitably cause side effects such as weight changes. Some previous studies have found that melatonin had no significant effect on human body weight, and the effects of different concentrations and durations of melatonin on body weight in different populations were also not consistent (Table 2). However, new evidence firstly raised by Delpino et al. showed significant results for exogenous melatonin in reducing body weight [23].

According to a recent systematic review and meta-analysis including 23 studies, 11 showed significant results from melatonin supplementation on weight loss, BMI, or waist circumference, compared with placebo, and the results were better in studies that used doses of ≤8 mg/d [23]. It was reported that once the standard treatment induces weight gain, melatonin can slightly reduce this effect, and vice versa and melatonin is more effective for children and adolescents [32]. Taken together, the present evidence appears to show that melatonin has potential in body weight reduction, whereas more studies with greater heterogeneity are needed to further confirm this effect in clinical. The dose, time, and duration of melatonin administration should be considered as a treatment option among the factors that determine its efficacy.

### 2.2. Lipid Profile

Ahmad et.al first reported a positive effect of melatonin on overweight and lipid profile of rats with obesity and diabetes [33]. Long-term melatonin administration can reduce weight gain and the serum total cholesterol (TC) levels [19], and inhibit the absorption and biosynthesis of cholesterol [34] as well as increasing its catabolism [35]. The similar results in male C57BL/6 mice [36], Wistar rats [37], and Syrian hamsters [38] fed a high-fat diet (HFD) had shown that melatonin significantly reduced the levels of serum triglyceride (TG), TC, and low-density lipoprotein- cholesterol (LDL-C). Besides, epidemiological evidence and Meta-analyses also support the improved effects of melatonin on serum lipid profile, and suggest the preventive role in cardiovascular disease [39,40,41], but not in menopausal women [42]. The hypocholesterolemic effect of melatonin works through the augmentation of endogenous cholesterol clearance mechanisms, via the synthesis of bilirubin acid and inhibition of low-density lipoprotein receptor activity [43,44]. Through increasing circulating irisin levels and enhancing fecal cholesterol excretion, melatonin exerts the hypolipidemic effect [19].

### 2.3. Glucose Metabolism

Melatonin has been confirmed to improve insulin sensitivity [45], induce β-cell regeneration in the pancreas [46], promote hepatic glycogen synthesis [47], thus reducing hyperglycemia in rodents. However, in contrast to the decreased effect in animals, melatonin leads to an increased risk of hyperglycemia in some human studies. It is generally accepted that melatonin impairs glucose homeostasis, because the much higher expression level of MTNR1B mRNA, encoding MT2, carried the common rs10830963 variant in human pancreatic islets [48].

**Figure 1:**
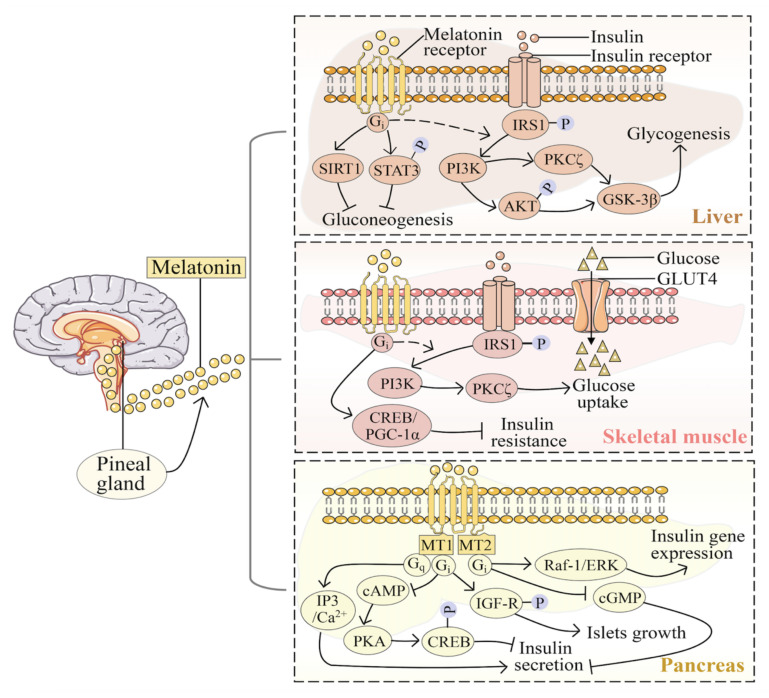
Melatonin signaling in glucose metabolic processes of the liver, skeletal muscle, and pancreas. AKT, protein kinase B; cAMP, cyclic adenosine monophosphate; cGMP, cyclic guanosine monophosphate; CREB, cAMP-response element-binding protein; ERK, extracellular signal-regulated kinase; GSK-3β, glycogen synthase kinase-3β; IGF-1, insulin-like growth factor 1 signaling; IRS1, insulin receptor substrate 1; MT1, melatonin receptor 1; MT2, melatonin receptor 2; PGC-1α, peroxisome proliferator-activated receptor-gamma coactivator 1-α; PI3K, phosphatidylinositol 3-kinase; PKA, protein kinase A; PKCζ, protein kinase Cζ; Raf-1, Ras-associated factor-1; SIRT1, silent information regulator 1; STAT3, signal transducer and activator of transcription 3.

Considering the expression of melatonin receptors in many tissues, studies have highlighted the influence of melatonin signaling in glucose metabolic processes of the peripheral tissue, such as liver [49], skeletal muscle [50], and pancreas [51]. Melatonin is essential for insulin-stimulated phosphatidylinositol 3-kinase (PI3K)–protein kinase B (AKT) activity [52]. In hepatocyte cells, melatonin mediated the glycogen synthesis via the insulin receptor substrate 1 (IRS1)–PI3K–protein kinase Cζ (PKCζ)–AKT–glycogen synthase kinase-3β (GSK-3β) pathway by the G_i_ protein [49]. In rats’ liver, melatonin promotes the expression of silent information regulator 1 (SIRT1) and phosphorylation of signal transducer and activator of transcription 3 (STAT3) to regulate gluconeogenesis [53]. Melatonin activates the IRS1-PI3K-PKCζ pathway to promote glucose uptake in mouse skeletal muscle [54]. It also activates the cyclic adenosine monophosphate (cAMP)-response element-binding protein (CREB)-peroxisome proliferator-activated receptor gamma coactivator 1-α (PGC-1α) pathway to prevent insulin resistance in rats [55]. In the pancreas, melatonin receptors couple with the multiple parallel signaling pathways to affect the different functions of insulin. In terms of the inhibition of insulin secretion, melatonin signals through Gi protein-coupled MT1 to inhibit cAMP-protein kinase A (PKA)-CREB pathway [56], or through Gi protein-coupled MT2 to inhibit the cyclic guanosine monophosphate (cGMP) pathway [57]. MT1 can also be selectively coupled with G_q_ protein to regulate the levels of inositol triphosphate and Ca^2+^ in cells, through which melatonin increases insulin secretion [58]. It activates the insulin-like growth factor 1 signaling (IGF-1) pathway via MT1 to regulate the growth and differentiation of islets [52]. Moreover, melatonin inhibits the expression of insulin genes through MT2 and the downstream Ras-associated factor-1 (Raf-1)-extracellular signal-regulated kinase (ERK) signaling pathway [59] (Figure 1).

### 2.4. Insulin Resistance

The role of melatonin in insulin resistance (IR) of peripheral tissues, including the adipose tissue [60], pancreas [61], and skeletal muscle [55] has also been addressed. Patients with obesity taking melatonin for 12 weeks show a pronounced decrease in the IR index [62]. In the case of existing IR, melatonin treatment improves glucose metabolism in the IR model by restoring the effect of insulin on the cardiovascular system [63]. Higher levels of endogenous nocturnal melatonin secretion are negatively related to the insulin level and onset of IR [64]. A link between the polymorphisms of the melatonin receptor genes and IR has also been brought to light [65]. Melatonin participates in improving IR may via MT1 [50] or by preventing mitochondrial dysfunction [55], promoting endoplasmic reticulum (ER) stress [64], improving hepatokines associated with insulin resistance and T2DM, such as alpha-2-HS-glycoprotein [56].

### 2.5. Prenatal Melatonin in Childhood Obesity

Maternal metabolic abnormalities are related to obesity in offspring, especially in childhood [66]. Many prenatal risk factors that lead to fetal metabolism, including gestational diabetes and night work, are related to the reduction of pineal gland-derived melatonin and related changes in the circadian rhythm [67]. A variant in the MTNR1B gene is associated with gestational diabetes mellitus in East Asian women [68]. Thus, women carrying the MTNR1B G allele are at higher risk of hyperglycemia, and increased glucose transfer to the placenta can lead to obesity in offspring. The maternal MTNR1B genotype interacts with pregnancy weight gain and affects the risk of childhood obesity in the offspring [66]. In addition, melatonin from breast milk influences weight gain in infants, limiting the development of obesity and complications in the long run [69]. Melatonin protects against maternal obesity-associated oxidative stress (OS) and meiotic defects in oocytes via the silent information regulator 1(SIRT3)-superoxide dismutase (SOD) 2-dependent pathway [70].

## 3. Effect of Melatonin on Obesity

### 3.1. Melatonin in Adipose Tissue

**Figure 2:**
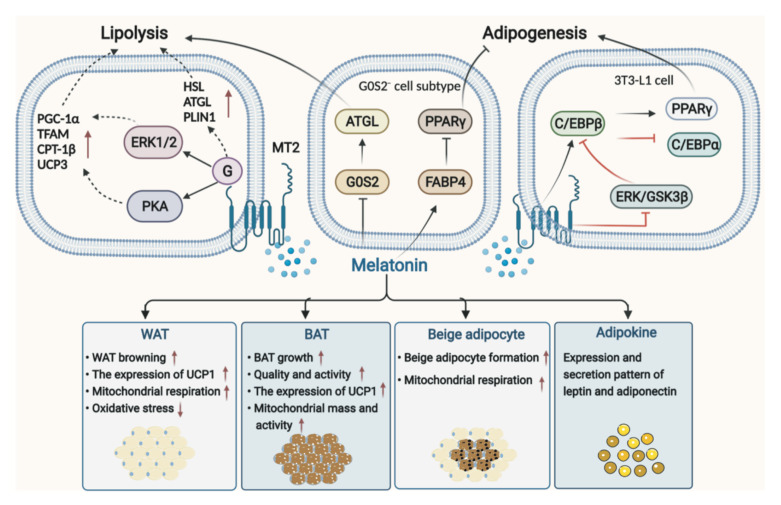
Mechanism of melatonin in lipolysis of adipocyte and adipogenesis, and its role in WAT, BAT, and beige adipocytes as well as adipokines. AKT, protein kinase B; ATGL, adipocyte triglyceride lipase; BAT, brown adipose tissue; C/EBP, CCAAT/enhancer-binding protein; CPT-1β, carnitine palmitoyltransferase-1β; ERK, extracellular signal-regulated kinase; GSK-3β, glycogen synthase kinase-3β; HSL, hormone-sensitive lipase (HSL); MT2, melatonin receptor 2; PGC-1α, peroxisome proliferator-activated receptor-gamma coactivator 1-α; PLIN1, perilipin 1; PPARy, peroxisome proliferator-activated receptor γ; TFAM, transcription factor A mitochondrial; WAT, white adipose tissue; UCP1, uncoupling protein 1.

The pathophysiological mechanism of obesity is complex, and obesity is characterized by severe dysfunction of white adipose tissue (WAT), including changes in its endocrine function [71]. Traditionally, adipose tissue in mammals is classified as WAT and brown adipose tissue (BAT) [72], with the former acting as energy storage and the latter acting as an energy consumer. Both BAT and beige adipose tissue formed by WAT browning contain abundant mitochondria and uncoupling protein (UCP) 1 that benefit for weight loss and energy-burning [73]. Thus far, it has been reported that melatonin can regulate adipose tissue and adipokines, such as lipolysis of adipocyte, fat deposition, BAT growth, beige adipogenesis, and WAT browning, which in turn affects energy expenditure [74]. Especially, the possible mechanisms of melatonin-mediated signaling pathways in lipolysis and adipogenesis have been well summarized by Pan et al. [75]. Melatonin could significantly induce lipolysis of adipocytes and up-regulate the expression of lipolytic genes and proteins via MT2, including hormone-sensitive lipase (HSL), adipocyte triglyceride lipase (ATGL), and perilipin 1 (PLIN1) [76]. Mediated by MT2 activating ERK1/2 and PKA pathway, melatonin significantly increased cellular respiratory capacity, upregulated the expression of PGC-1α and transcription factor A mitochondrial (TFAM), increased mitochondrial copy number, and induced the robust expression of thermogenic genes in intramuscular preadipocytes, including carnitine palmitoyltransferase-1β (CPT-1β) and UCP3, and triggered differentiation toward beige phenotype genes, cell death-inducing DFFA-like effector A (CIDEA) and Prdm16 [77]. However, the contribution of melatonin to the regulation of adipogenesis remains uncertain [76]. Some studies demonstrated that melatonin suppressed adipogenesis by down-regulating peroxisome proliferator-activated receptor γ (PPARγ), CCAAT/enhancer-binding protein (C/EBP) β, and C/EBPα in 3T3-L1 cells [78]; however study also showed that melatonin stimulated adipocyte differentiation in 3T3-L1 cells and increased intracytoplasmic TG accumulation in murine fibroblasts by up-regulating PPARγ, C/EBPα, and C/EBPβ [79]. These results depend to some extent on the melatonin concentration and exposure time as well as the cell type [80]. Notably, a recent study using Single-cell RNA sequencing of preadipocytes found that melatonin induced pre-adipocyte heterogeneity, producing a G0S2− cell subtype, which is of great benefit for promoting lipolysis and inhibiting adipogenesis. Melatonin plays this role by down-regulating G0S2 in the G0S2− cell subtype and thus leads to activation of adipose triglyceride lipase (ATGL), or by up-regulating fatty acid-binding protein 4 (FABP4) in the G0S2− cell cluster and leads to inhibition of PPARγ, further reducing adipogenesis [81] (Figure 2).

#### 3.1.1. Melatonin in WAT

Melatonin has remarkable effects on WAT, including stimulating WAT browning and beige adipocyte formation, improving mitochondrial function, and relieving OS. It was reported that long-term melatonin treatment drove WAT into a brown-fat-like function and induced beige formation in ZDF rats, along with upregulation of UCP1 expression, which contributed to thermogenesis and weight control [82]. In addition, melatonin is capable of improving mitochondrial respiration in WAT and beige adipocytes as well as reducing OS [83]. In conclusion, the role of melatonin in WAT browning may be related to its effects against OS, uncoupling the mitochondrial bioenergetic process by enhancing the expression of UCP-1 [84].

#### 3.1.2. Melatonin in BAT

The activity of BAT in energy expenditure provides potential therapeutic prospects for counteracting obesity. In particular, the role of melatonin in stimulating BAT growth, enhancing BAT quality and activity, improving mitochondrial function and activities, and increasing UCP1 expression, as well as decreasing oxidative and nitrosative stress and susceptibility of adipocytes to apoptosis [85], make it an essential way to burn energy. Melatonin can regulate the physiology of BAT, which not only increases the recruitment of BAT cells but also enhances metabolic activity in mammals [14]. Melatonin could increase the quality of BAT in Zucker rats with diabetes and obesity [86] and improved aged rats’ BAT thermogenic potential in the cold acute challenge [17]. Pinealectomy led to reduced acute thermogenic capacity [87]; however, melatonin treatment reversed it and increased the expression of key genes such as UCP1, indicating that melatonin affected the thermogenic activation pathway [82]. Moreover, in a small-scale human study, patients with pineal gland resection due to pineal tumors who were treated with melatonin showed increased BAT volume and activity [88]. Melatonin affects BAT possibly via the following four aspects. Firstly, the effects of melatonin on BAT may be mediated by membrane melatonin receptors located both centrally and peripherally. Melatonin stimulates MT1 located on neurons of the hypothalamus and acts on SCN to increase noradrenaline turnover, gene expression of UCP1, PPARγ, PGC1 in BAT, and promote BAT function in nonshivering thermogenesis [14]. Secondly, melatonin may act directly on BAT. Melatonin reduced intracellular cAMP, which subsequently affected PKA activity and phosphorylation of cAMP-response element-binding protein, and upregulated the expression of UCP1 via activation of MT1 and MT2 in BAT [14,73]. Third, the non-shivering thermogenesis function of brown adipocytes could be improved by melatonin and its metabolites’ synergistic effect at the mitochondrial level. It was demonstrated that melatonin improved both the content of mitochondria and the thermogenic function of BAT [14,84]. In addition, the interaction between melatonin and glucocorticoid, prolactin, insulin, glucagon, especially leptin and thyroid is also crucial to BAT [14].

#### 3.1.3. Melatonin in Adipokines

Leptin and adiponectin produced by adipocytes are the major adipokines relating to the pathogenesis of obesity. Surprisingly, the oral melatonin administration positively regulated the leptin level [89,90]. A lack of melatonin signaling induced leptin resistance, suggesting a vital role of melatonin in leptin signaling [91]. In mice with obesity, melatonin reduced adipocyte hypertrophy and inversely regulated the expression of adiponectin [92]. Melatonin contributes to normalizing the expression and secretion patterns of the two adipokines [93], which provides a broader perspective for the relationship between melatonin and obesity. Melatonin and the two adipokines work through parallel signaling pathways, reciprocally disturbing the effects they induce in organisms, in which time, biology, and the circadian system are strongly linked to obesity seem to play a special role [94].

### 3.2. Melatonin in Liver

The liver is associated with lipid digestion, absorption, transportation, and catabolism, and liver glycogen plays an important role in regulating blood glucose concentration to maintain its stability. The role and pathway of melatonin in maintaining liver glycogen synthesis have been described above. Considerable evidence has shown the protective role of melatonin in liver function in HFD, NAFLD, T2DM, and liver fibrosis. Based on a recent meta-analysis, melatonin supplementation could improve liver enzymes such as aspartate aminotransferase, alkaline phosphatase, and gamma-glutamyltransferase, in patients with NAFLD [95]. Melatonin markedly decreased activities of the hepatic lipogenic enzymes, including SREBP1c, fatty acid synthase (FAS), stearoyl-CoA desaturase 1 (SCD1), acetyl-CoA carboxylase (ACC) and PPARγ [96], and elevated the relative hepatic carnitine palmitoyltransferase-1α expression in HFD-induced hyperlipidemia [38]. The crucial role of melatonin in the development of NAFLD, probably via mitogen-activated protein kinase (MAPK)/c-Jun N-terminal kinase (JNK) signaling [97] or elevating activation of apoptosis signal-regulating kinase 1 (ASK1) and downstream signaling pathways to decrease de novo lipogenesis in the liver [98], and via the nuclear receptor subfamily, 4 group A member 1 (NR4A1)/DNA-PKs/p53 pathway [99]. Furthermore, melatonin reverses the loss of mitochondrial respiratory function, blocks cell oxidative damage, and reduces calcium overload under HFD, thus protecting mitochondrial division and mitochondrial autophagy [99].

### 3.3. Melatonin in the Pancreas

The pancreas’s internal and external secretory function makes it crucial in regulating blood glucose, and digesting glucose and fat. Treatment with melatonin significantly mitigates pancreatic injury, including impairment of exocrine and endocrine pancreatic functions [100]. As mentioned above, melatonin affects the secretion of insulin by β cells, and it also affects glucagon secretion by α cells, with the involvement of melatonin’s modulation of PI3K, intracellular messengers like cAMP, cGMP, arachidonic acid, and calcium ions in various condition, and regulation of the glucagon promoter by melatonin-induced activation of phospholipase C (PLC) [57]. The role of melatonin in pancreatic protection involves complex mechanisms, such as inhibiting OS and ER stress, reducing pro-inflammatory cytokines and prostaglandins, activating heat shock proteins, reducing pancreatic necrosis, and increasing regeneration [101].

### 3.4. Melatonin in Skeletal Muscle

Considering that skeletal muscle metabolism can also affect glucose and lipids metabolism, the interaction between melatonin and skeletal muscle metabolism also throws new light on the mechanism of melatonin in obesity. As summarized by Genario R et al., muscle mass can prevent obesity and other metabolic disorders, including sarcopenia obesity, and melatonin could positively affect myocyte metabolism [10,102]. There is an inverse association between urine melatonin and sarcopenia [103], and melatonin therapy can slow down muscle atrophy [104], suggesting the protective role of melatonin in skeletal muscle. The antioxidant effect of melatonin counteracts mitochondrial impairments and reduces OS and autophagic alterations in muscle fibers, which benefits for restoring muscle decline [105]. Besides, the regulatory role of the melatonin nexus linking muscle regeneration and repair, proliferation, differentiation, and myofiber formation as well as the treatment of muscular diseases has been discussed. Melatonin is suggested to alleviate myofiber size, mitochondria fusion, cristae preservation, and satellite cells in skeletal muscle disorders by inhibition of megamitochondria, reactive oxygen species (ROS) production, inflammation as well as apoptosis [106]. Although melatonin can protect skeletal muscle, the specific mechanism especially the process of muscle differentiation and fusion is still unclear [107].

## 4. Potential Mechanisms of Melatonin in Obesity

### 4.1. Melatonin Receptors

Melatonin membrane receptors, MT1 and MT2, are widely expressed in various tissues, and melatonin achieves actions by directly or indirectly interacting with them [108]. In addition to activating the most widely studied G_i_/_o_-cAMP and its downstream effector factors (including PI3K, AKT, ERK, etc.) pathways, MT1/MT2 also activates the G_q_/_11_-PLC-Ca^2+^ pathway and recruit β-arrestins [109]. Both receptors generally interact with the cAMP/PKA or G_i_-ERK pathway with quite different downstream pathways [110]. MT1/MT2 has been widely studied in glucose metabolic disorders. The polymorphism of the MTNR1B locus encoding MT2 in humans has a strong correlation with impaired insulin secretion and elevated fasting blood glucose [111]. Clear evidence has also shown the critical role of MT1 in regulating glucose homeostasis in rodents. The mechanisms whereby melatonin regulates physiological metabolism can be revealed by a series of studies with melatonin receptor knockout mice. [112]. Mice with MT1 removed caused systemic IR by modulating the activity of PI3K [50]. The phase of the mouse circadian clock was advanced by MT1-selective inverse agonists when given at subjective dusk, and the agonist-like effect was eliminated in MT1 [113]. A recent study has also highlighted the effective role of melatonin receptor MT1/MT2 agonist, ACH-000143, in weight loss and liver TG levels reduction in the HFD rat model [114]. Furthermore, MT3, which is also called chinone reductase 2, inhibits the generation of ROS in mitochondria by preventing the entry of reducing equivalents from cathinone into the electron transport chain. Melatonin binds to the MT3 binding site and the cytosolic enzyme chinone reductase 2, thus scavenging free radical [115]. In addition to membrane receptors, the melatonin nuclear receptor retinoid Z receptor α (RORα/RZR) also plays an important role in the function of melatonin [116], which regulates the biological clock circuitry and play a key role in the integration of circadian outputs and metabolic processes [117].

### 4.2. Circadian Rhythm

Over the years, the chronobiological study has raised awareness of the critical role of the circadian rhythm in obesity. The circadian system exists to synchronize physiology and behavior with a 24-h environmental cycle and optimize energy balance [118], thereby regulating the daily rhythm of several physiological and behavioral processes, such as eating/fasting and waking/sleeping [119]. Circadian disturbances due to shifting work [120], continuous light exposure [121], and dietary changes [122] can adversely affect energy balance and increase the risk of weight gain.

**Figure 3:**
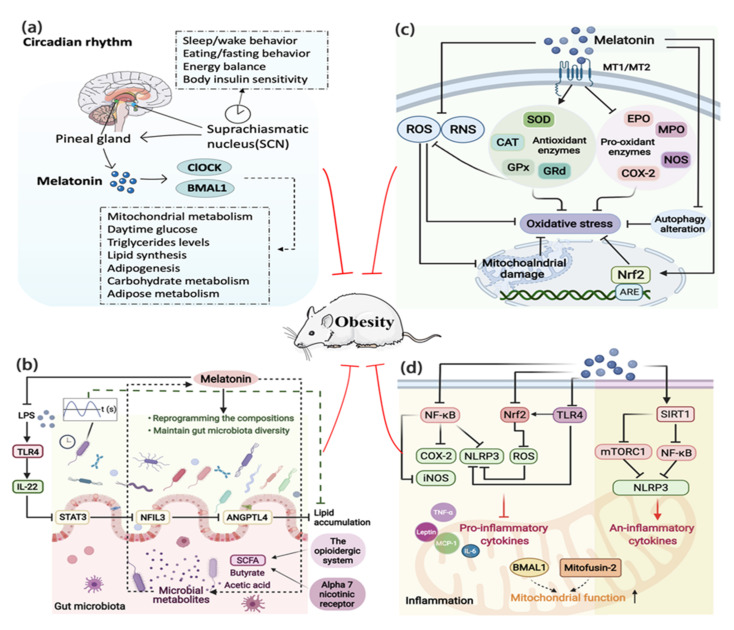
The potential ways by which melatonin regulates obesity. (**a**) Melatonin’s secretion is controlled by the SCN, which in turn affects the main clock, SCN. Melatonin is key for the modulation of CLOCK and BMAL1 that regulate the processes necessary for energy balance, such as mitochondrial metabolism, daytime glucose, lipid synthesis, adipogenesis, and carbohydrate metabolism, etc. (**b**) Melatonin administration can not only reprogram the composition of gut microbiota and maintain gut microbiota diversity but also improve its diurnal rhythms. The interactions of melatonin with microbial metabolites, short-chain fatty acids, such as butyrate and acetic acid, balance the energy homeostasis. Via inhibited LPS induced TLR4 signal pathway, melatonin can reduce lipid accumulation. (**c**) Melatonin directly or indirectly inhibits oxidative stress or mitochondrial damage by directly inhibiting the production of ROS and RNS, promoting antioxidant enzymes, inhibiting pro-oxidant enzymes and autophagy. (**d**) Melatonin inhibits inflammation via inhibition of NF-κB, NRF2, TLR4, and SIRT1 signal pathways, thus leading to the downregulation of pro-inflammatory cytokines and upregulation of anti-inflammatory cytokines. ANGPTL4, angiopoietin-like 4; ARE, antioxidant response element; CAT, catalase; COX-2, cyclooxygenase-2; EPO, eosinophil peroxidase; Gpx, glutathione peroxidase; GRd, glutathione reductase; IL-6, interleukin-6; IL-22, interleukin-22; iNOS, inducible nitric oxide synthase; LPS, lipopolysaccharide; MCP-1, monocyte chemotactic protein-1; MPO, myeloperoxidase; mTORC1, mTOR complex 1; NFIL3, nuclear factor interleukin-3-regulated protein; NF-κB, nuclear factor κB; NLRP3, NLR family pyrin domain-containing 3; NOS, nitric oxide synthase; Nrf2, nuclear factor erythroid 2-related factor 2; RNS, reactive nitrogen species; ROS, reactive oxygen species; SCFA, short-chain fatty acid; SIRT1, Sirtuin-1; SOD, superoxide dismutase; STAT3, signal transducer and activator of transcription 3; TLR4, toll-like receptor 4; TNF-α, tumor necrosis factor-α.

Known as the powerful endogenous synchronizer of the circadian rhythm, melatonin is a key factor that links the circadian rhythm to lipid metabolism [112]. Melatonin is pivotal for coordination between the environment and the circadian distribution of physiological and behavioral processes necessary for energy metabolism and weight gain, including synchronization of the activity-feeding/rest-fasting cycle, restoration of insulin sensitivity, and loss of glucose tolerance [11]. It was indicated that melatonin may act on the synchronization of clock genes to prevent the desynchronization caused by the HFD intake [36]. The circadian effect of melatonin is mediated by the main circadian genes ClOCK and BMAL1, which are crucial for the circadian regulation of mitochondrial metabolism and wider energy regulation, such as daytime glucose, TG levels, lipid synthesis, lipogenesis, carbohydrates, and lipid metabolism [67,123] (Figure 3a). Environmental disruption of the circadian rhythm induces or accelerates obesity development; however, melatonin reverses this situation [124,125]. Irregular light conditions, one of the most important factors affecting the biological clock, are becoming a new harmful environmental factor for weight gain and obesity. A study based on 10 healthy young men showed that acute blue light exposure before bedtime aggravated the circadian rhythm, inhibited the fat oxidation, and suppressed the level of melatonin, suggesting that long-term blue light exposure at night may cause obesity due to circadian rhythm and macronutrient metabolism disorders risk [126]. While constant light exposure brought negative effects on weight gain, IR, and intestinal gene expression of the circadian clock in mice, melatonin coordinated lipid homeostasis by restoring intestinal circadian gene expression and ameliorating rhythm disorder [127]. In addition to the gut clock, the circadian rhythm controls many other peripheral clocks, including adipose tissues clock [128]. Melatonin promoted adipocyte proliferation by forming a Clock/histone deacetylase 3 (HDAC3)/c-Myc complex and subsequently driving the circadian amplitudes of proliferation genes, revealing a novel mechanism that links circadian rhythm to cell proliferation in adipose tissue [129].

Obesity is a well-known risk factor for infertility, which is also a bad outcome of circadian rhythmicity disruption. Previous studies have confirmed that melatonin can regulate the hypothalamic-pituitary-gonadal axis to influence reproductive functions [130]. It was reported that melatonin supplementation would reduce obesity-related spermatogenic and steroidogenic dysfunctions induced by HFD [131]. Up to now, researchers have established the connection between clock genes, hormones, and obesity-related reproductive processes. Changes in the follicle-stimulating hormone, luteinizing hormone, and prolactin levels are accompanied by sleep disorders or circadian rhythm disturbances. The circadian rhythms disrupted by shift work, jet lag, and light are related to lower fertility rates and early pregnancy outcomes. The circadian rhythm modulates the circadian gene, CLOCK, to regulate the decline in fertility and the increase in abortion rate [132]. It was indicated that chronic high-temperature exposure is an environmental stimulus that disrupts the circadian rhythm, which will affect the normal rhythmic oscillation of serum steroid hormones and the expression of testicular clock genes and genes involved in steroid generation [133]. Sciarra et al. had well demonstrated the relationship between the fertility-related hormones and circadian rhythms, including melatonin, gonadotropins, estrogens, androgens, and glucocorticoids. Clock genes affect infertility, produce low levels of sex hormones, cause embryo implantation failure, and reduce newborn size in mouse models and shift-working women [134].

Interestingly, Cryptochromes, a kind of blue light-sensitive receptor [135], are represented as the major chronobiology like melatonin. Because blue light is the strongest synchronizing agent of the circadian rhythm system that keeps most of the internal functions of physiology in sync, it is the most important wavelength to inhibit the secretion of endogenous melatonin [136]. Except for the MTNR1B genes discussed above, variations in the Cryptochrome 1 and Cryptochrome 2 genes are also related to both fasting blood glucose and the circadian clock. Yoshiuchi firstly observed genetic population differences and ethnic diversity in humans at one glucose-associated Cryptochrome 1 single-nucleotide polymorphism (SNP) (rs8192440), one glucose-associated Cryptochrome 2 SNP (rs11605924), and one glucose-associated MTNR1B SNP (rs10830963), which reflected that the evolution of the three biological clock-, energy consumption- and glucose-related genes may represent the historical impact of environmental pressure on the human genome [137].

### 4.3. Involvement of the Gut Microbiota

As an important environmental signal, the gut microbiota is important for absorbing nutrients and maintaining metabolism [138], which makes it another important hub in the pathophysiology of obesity. Melatonin is a hormone produced both by the host and microbiota, and its receptors have been widely found in intestinal tissue [139]. Melatonin has been confirmed to improve gut microbiota dysbiosis in colitis induced by sleep deprivation [140] or in a weanling mouse model [141]. Interestingly, melatonin can improve the impaired composition of gut microbiota and maintain gut microbiota diversity in HFD-fed mice [142,143]; more specifically, melatonin promoted a decrease in the ratio of Firmicutes to Bacteroidetes and an increase in the relative abundance of Akkermansia while normalizing the richness and diversity of Alistipes, Anaerotruncus, and Desulfovibrionaceae, thereby alleviating weight gain, liver steatosis, IR, and low-grade inflammation [21]. The underlying mechanism may partly involve the circadian transcription factor, nuclear factor interleukin-3-regulated protein (NFIL3), which can be activated by gut microbiota and regulate lipid absorption and export in intestinal epithelial cells [144]. As reported, oral melatonin decreased the quantity of E Coli-generated lipopolysaccharide (LPS), which alleviated NFIL3-induced transcriptional inhibition of angiopoietin-like 4 (ANGPTL4) through the toll-like receptor 4 (TLR4)/interleukin-22 (IL-22)/STAT3 signaling in the ileum, thereby ameliorating ileal lipid intake and lowered fat accumulation in epididymal-WAT [145]. Besides, as the gut microbiota exhibits rhythmicity in a light/dark cycle, it can serve as a potential mechanism for the circadian clock-lipid metabolism interplay [112]. Melatonin was proved to ameliorate the diurnal rhythms or rhythmic disorders of the gut microbiota, which contributed to improving gut microbiota dysbiosis and promoting lipid efflux from the intestine [127,146]. Furthermore, the interaction between melatonin and microbial metabolites, especially short-chain fatty acid (SCFA) such as butyric acid, may also be a potential channel through which melatonin regulates energy metabolism. The effects of butyrate are mediated in part by increasing the melatonergic pathway, which suggests that the gut microbiota interacts with melatonin. Part of melatonin’s effects appear to be mediated via α-7 nicotinic receptors, and both melatonin and butyrate may regulate obesity through the opioid system [67]. Melatonin can also prevent lipid metabolic disorders via a mechanism of the microbiota-acetic acid axis, especially in the alterations of Bacteroides and Alistipes abundances [143]. Meanwhile, the gut microbiota will affect muscle composition and metabolism, and the concept of the gut–muscle axis has been formulated. The role of melatonin in the gut microbiota makes melatonin a promising therapeutic agent to limit muscle deterioration [106]. To conclude, melatonin works through the gut microbiota to defense against obesity, particularly involving the circadian rhythm, skeletal muscle, and its metabolites (Figure 3b). Surprisingly, Liu et al. recently proposed that intermittent fasting could reshape the intestinal microbiota and metabolome, reducing weight gain in mice more effectively than melatonin monotherapy. There was almost no interaction between intermittent fasting and melatonin except for the effect on the area and number of adipocyte area and number, the abundance of Bacteroides and Akkermansia, as well as the intestinal metabolites alanine, valine, and isoleucine [20]. Nevertheless, it still cannot deny the beneficial effects of melatonin in reshaping or improving the gut microbiota.

### 4.4. Melatonin and Sleep Disorders

Lack of sleep is a known risk factor for metabolic diseases, including obesity [147], T2DM [148] and heart disease [149]. The link between sleep restriction (SR) and obesity has been well established, involving the mechanisms of the negative impact of SR on appetite and food intake regulation, altered thermoregulation, increased fatigue, and lower physical activity level [150]. Referred to as the hormone of darkness [151], melatonin has been approved by the European Medicines Agency for primary insomnia in adults over 55 years of age since 2007 [152], and dual melatonin receptor agonists are being trialed in various sleep disorders [153]. Melatonin acts on SCN to weaken the wake-up signal of the circadian clock, thereby promoting sleep [154]. Besides, through reducing the activation of the DMN (precuneus), melatonin improves sleep quality in patients with insomnia and benefits cardiovascular health [155]. Studies have also highlighted that melatonin may act via the MT1 to inhibit orexin neurons and promote sleep [156]. Predictably, the beneficial effect of melatonin on improving sleep may be another breakthrough in regulating metabolic diseases such as obesity. First of all, melatonin influences the orexigenic and anorexigenic neurons and neuropeptides thereby regulating appetite and energy expenditure [90]. Since orexin is the center of the hypothalamus that regulates diet and energy balance, melatonin inhibits orexin and further regulates sleep, making it a potential molecule for regulating obesity. Secondly, melatonin is closely related to the disturbance of sleep disturbance induced by gut microbiota disorders. It was reported that supplementation with 20 and 40 mg/kg melatonin reversed sleep deprivation-induced dysbiosis of the microbiota in the colon [140]. The level of melatonin in children with obesity increased during 1 h of sleep, which was up-regulated in the context of obesity as a compensatory mechanism. The organism triggered its production to increase drowsiness, promote behaviors that increased sleep time or counteract the pro-inflammatory and antioxidant effects caused by obesity and lack of sleep [157]. Therefore, the improvement of sleep disorders by melatonin may be of great significance in the regulation of obesity.

### 4.5. Melatonin and Oxidative Stress

Various pro-inflammatory cytokines generated by the adipose tissue produce ROS and induce OS [158], which play a crucial role in the occurrence of obesity. Since melatonin was determined to be an effective free radical scavenger and natural antioxidant, the concept that melatonin protects against OS under a remarkably large number of circumstances has been widely accepted [159]. Evidence of melatonin ameliorating OS has been found in both animals and humans with obesity [41,160,161]. Melatonin could reverse the adverse effects of obesity in perivascular adipose tissue that included overproduction of ROS, reduced superoxide dismutase activity, and decreased bioavailability of NO [162]. Melatonin also stimulated the SIRT1/nuclear factor erythroid 2-related factor 2 (Nrf2) signaling pathway to reduce lipopolysaccharide-induced ROS generation [163], and it may ameliorate hydrogen peroxide (H_2_O_2_)-induced OS through modulation of ERK/AKT/nuclear factor κ (NF-κB) pathway [164].

The powerful antioxidant function makes it possible for melatonin to fight obesity. Based on the reported evidence, the underlying mechanism can be explained as follows (Figure 3c): firstly, melatonin can directly scavenge ROS under OS conditions, such as H_2_O_2_ and superoxide anion [165]. The direct action of melatonin is dependent on the buffering capacity of its aromatic indole ring reacting with ROS or reactive nitrogen species (RNS), which leads to the formation of metabolites that in turn exhibit antioxidant function through a cascade reaction mechanism [12,166]. Secondly, melatonin indirectly scavenges ROS by activating antioxidant enzymes related to glutathione metabolism, protecting tissues such as the pancreas, adipose tissue, and liver from OS [162,167], through which it inhibits mitochondrial damage. The indirect antioxidant action involves the activation of MT1 and MT2, which stimulates the expression and activity of endogenous antioxidant enzymes including SOD, catalase (CAT), glutathione peroxidase (GPx), and glutathione reductase (GRd). Melatonin also protects antioxidant enzymes from oxidative damage and increases the synthesis of GSH [12]. Thirdly, melatonin increases antioxidant defenses by epigenetically inducing Nrf2, which binds to antioxidant response elements (AREs) [71]. Melatonin can also suppress the activity of pro-oxidant enzymes including myeloperoxidase, eosinophil peroxidase, nitric oxide synthase, and cyclooxygenase-2 (COX-2) [168,169]. In addition, melatonin can chelate transition metals, which are involved in the Fenton/Haber-Weiss reactions, and hence reduce the formation of the devastatingly toxic hydroxyl radical resulting in the reduction of OS [168]. Another mechanism is due to melatonin’s ability to support the electron flux through the respiratory chain, preventing the breakdown of the mitochondrial membrane potential, and decreasing electron leakage, thereby reducing the formation of superoxide anions [170]. Furthermore, melatonin can regulate autophagy, an intracellular degradation pathway that closely relates to obesity [171]. Melatonin treatment improved possible oxidative homeostasis through autophagic induction [172], by directly modulating its activity and improving the proteolytic pathway as well as by indirectly reversing mitochondrial damage due to the inhibition of OS [173].

### 4.6. Melatonin and Inflammation

Obesity is widely recognized as a metabolic disease characterized by chronic low-grade inflammation [174]. As an anti-inflammatory agent, melatonin can promote the recovery of some chronic diseases by improving the inflammatory response [175]. Melatonin is shown to attenuate the inflammatory response in the brain and peripheral tissues in patients with obesity [176,177]. Above all, melatonin relieves inflammation and subsequently improves the systemic inflammatory state following obesity [178], mainly owing to its role in the upregulation of anti-inflammatory cytokines and downregulation of pro-inflammatory cytokines [179], such as leptin, interleukin-6 (IL-6), monocyte chemotactic protein-1 (MCP-1), and tumor necrosis factor-α (TNF-α) [22,36]. The impact of melatonin in downregulating pro-inflammatory cytokines can lead to multiple antioxidant functions [109], ROS inhibition, downregulation of neuronal NO synthases and COX-2, upregulation of Nrf2, inhibition of inflammasome NLR family pyrin domain-containing 3 (NLRP3), and NF-κB activation [180]. For example, melatonin alleviated NF-κB and NLRP3-inflammasome signals and thus led to the inhibition of GSDMD cleavage and pyroptotic cell death in the adipose tissue of obese mice [181]. The mainstream view is that the anti-inflammatory effect of melatonin is mainly mediated by inhibiting the formation of NLRP3 inflammasomes [182]. Secondly, the anti-inflammatory actions of melatonin may involve the upregulation of SIRT1, which shares common effects with melatonin [180]. The increased expression of SIRT1 regulated by melatonin inhibits the activation of NF-κB [183] and the NLRP3 inflammasome [184]. It has been confirmed that melatonin prevented lipotoxicity through modulating SIRT1 and in turn mitochondria signaling, thus reducing OS and inflammation [185,186]. Thirdly, the anti-inflammatory action of melatonin relates to its activity as an optimizer of mitochondrial function. Melatonin can improve mitochondrial respiration [187] and retain the activity of complexes I and III, inhibit the opening of mitochondrial permeability transition pores and the release of cytochrome c [188,189]. These actions may work directly or through BMAL1, partly through the disinhibition of the pyruvate dehydrogenase complex and thus results in an increase in acetyl-CoA, which is a necessary co-substrate for activation of the mitochondria melatonergic pathway, allowing melatonin to optimize mitochondrial function [190]. To highlight, melatonin can produce beneficial effects on mitochondrial by mitofusin-2 that modulates the orexigenic agouti-related protein neuronal activity and diet-induced obesity, and the intrinsic apoptotic cascade modulation [191] (Figure 3d). Notably, the potential improvement of melatonin in obesity-related immune functions has also shed new light on this field. In the context of COVID-19, studies have revealed the potent antioxidant with immunomodulatory action and anti-inflammatory effects of melatonin in individuals with obesity and diabetes with the coexistence of COVID-19 [192]. Recently, Pivonello et al. highlighted the key role of melatonin as an entrainer of metaflammation and infections in obesity, addressing that melatonin could regulate the immune system by directly acting on the morphology and activity of the thymus, as well as regulating OS and inflammation during infection. Besides, the close connection between melatonin and immune response regulation is coordinated by TLRs, whose signaling would be strongly inhibited by melatonin [182]. Therefore, the role of melatonin in improving inflammation response provides insights for defending against obesity.

### 4.7. Other Related Ways

Firstly, melatonin effects may be via its induction of the α7nAChR. Melatonin’s positive regulation of the α7nAChR may also directly regulate obesity since α7nAChR agonism modulates the activity of hypothalamic neurons involved in food intake regulation [193]. Secondly, alterations in the opioidergic system, especially via reward regulation, are intimately associated with food intake and its dysregulation in obesity [194]. A growing body of evidence indicates the prominent actions of melatonin on the opioidergic system [195]. Melatonin act via the regulation of the opioidergic system and therefore with the subjective pleasure and dysphoria that drive food intake [67]. The effects include positively regulating the circadian levels of β-endorphin, the endogenous μ-opioid receptor agonist, as well as decreasing κ-opioid receptor levels [196]. Thirdly, recent studies have addressed the relationship between exosomes and melatonin in obesity [197]. Melatonin can increase the level of exosomal α-ketoglutarate derived from the adipose tissue, the target for the inhibition of melatonin-mediated adipose inflammation [177]. Besides, melatonin-stimulated exosomes originating from adipose-derived mesenchymal stem cells inhibit the inflammatory response, by transferring exosomal miRNAs including miR-34a, miR-124, and miR-135b [198].

## 5. Clinical Safety of Melatonin

Given the increasing frequency of melatonin usage in both clinical and daily life, the safety of melatonin in humans has been largely investigated. Inspiringly, many experiments and clinical studies have provided useful information about the safety and effectiveness of melatonin alone or as a complementary treatment. In adults, short-term use of melatonin is safe for adults, except for pregnant and lactating women who lack clinical data. There is no research showing that exogenous melatonin can cause any serious side effects, and it has only mild side effects such as reports of dizziness, headache, nausea, increased nocturnal enuresis, morning drowsiness [199,200]. In clinical studies on children, taking melatonin in the short and medium-term also produced mild side effects [12]. According to Rzepka-Migut et al.’s work based on multiple clinical results, the clinical side effects of melatonin for pediatric patients are also minor [201]. A report of 387 people included in 7 trials pointed out that only two cases reported serious adverse events in children and adolescents, such as migraine and mild generalized epilepsy, claiming that melatonin is an effective and tolerable drug for short-term treatment of sleep insomnia in children and adolescents [202]. During the 104-week treatment period, the optimal dose of nightly pediatric prolonged-release melatonin (2, 5, or 10 mg per night) was safe and effective for the long-term treatment of children and adolescents with autism spectrum disorder and insomnia. The most common treatment-related adverse events were fatigue (6.3%), lethargy (6.3%), and mood swings (4.2%). No harmful effects on child growth and puberty development had been observed [203]. Besides, surgery patients had psychomotor disorders, sedation, disorientation, and amnesia. Critically ill patients had mild headaches, increased sleepiness, and skin rashes, and the elderly had daytime sleepiness after taking melatonin [199]. Furthermore, rectal and vaginal melatonin could be used as a related alternative to standard oral melatonin therapy, and transdermal administration had a strong absorption capacity. Melatonin administered through these routes is safe [204]. In general, present clinical data show no serious side effects of exogenous melatonin administration.

## 6. Conclusions

Melatonin exerts beneficial effects on regulating lipid profile, insulin resistance, and maternal obesity, which may be owing to its role in regulating the adipose tissue, circadian rhythm, gut microbiota, sleep disorders, OS, inflammation, and others, such as α7nAChR and the opioidergic system. These mechanisms of action are interactive rather than completely independent. However, not all preclinical data show evidence of melatonin in weight loss, and clinical data is insufficient, which makes the role of melatonin in obesity a controversial issue. So far, there is no consensus on the possible role of melatonin as an auxiliary drug for the treatment of metabolic diseases, though it does show great potential in many aspects.

In the future, clarifying melatonin’s role in obesity will have to tackle some obstacles, especially the supplement of clinical data. Primarily, the clinical effectiveness of melatonin in obesity needs to be uncovered. Secondly, the physiological differences between diurnal humans and nocturnal rodents highlight the need for further consideration when extending rodent outcomes to humans. Moreover, the restricted number of participants, inconsistencies in application protocols, and numerous confusing environmental parameters limit intervention studies and, in most cases, even result in defective melatonin secretion profiles [109]. Thus, further research should combine larger clinical trials, higher doses of melatonin, longer study periods, and more comprehensive methods as well as account for circadian rhythms such as shift work, light exposure at night, and constant light exposure. Most importantly, further investigation into unraveling pathways in which melatonin participates in the process of obesity is required to better understand the mechanisms of melatonin in obesity.

## Figures and Tables

**Table 1:** Effect of melatonin administration on body weight in animals.

Subjects	Diet	Melatonin Administration	Body Weight	Other Related Effects	References
Female ICR mice	Normal	100 μg/mL/day in drinking water for 43 weeks	Decrease	Decrease abdominal fat deposition	Tamura et al. [15]
ICR mice	High-fat/ high-sugar	2.5–10 mg/kg/day in diet for 7 weeks	Decrease	Decrease levels of glucose, insulin, leptin	Onaolapo et al. [16]
Aged Wistar rats	Normal	10 mg/kg/day in drinking water for 16 weeks	Minor increase	Increase the thermogenic ability	Mendes et al. [17]
Sprague- Dawley rats	Normal	10 mg/kg/day by gavage for 12 weeks	Decrease	Decrease adipose deposition	Wang et al. [18]
Sprague- Dawley rats	High-fat	10 or 50 mg/kg/day in drinking water for 8 weeks	Decrease	Reduced serum TG	Tung et al. [19]
C57BL/6J mice	Normal	10 mg/kg/day in drinking water for 15 weeks	No effects	Improve intestinal *Allobaclum* content	Liu et al. [20]
C57BL/6J mice	High-fat	50 mg/kg/day by gavage for 10 weeks	Decrease	Decrease WAT weight	Xu et al. [21]
C57BL/6 mice	High-fat	1 mg/kg/day in water for 10 weeks	Decrease	Decrease fat deposition and adipocytes size	Farias et al. [22]

Abbreviations: TG: total cholesterol; WAT: white adipose tissue.

**Table 2:** Effect of melatonin administration on body weight in humans.

Subjects	Therapeutic Drug and Its Effect on Weight	Melatonin Administration	Comparison	Improving Effect of Melatonin	References
Adolescent patients with bipolar disorder	Olanzapine and lithium carbonate (weight gain)	3 mg/day for 6 or 12 weeks	Placebo	Reduce weight gain	Mostafavi et al. [24]
Patients with bipolar disorder or schizophrenia	SGAs (weight gain)	5 mg/day for 8 weeks	Placebo	Reduce weight gain	Romo-Nava et al. [25]
Adult patients with schizophrenia	Olanzapine (weight gain)	3 mg/day for 8 weeks	Placebo	Reduce weight gain	Modabbernia et al. [26]
55 patients with NAFLD	/	6 mg/day (1 h before sleep) for 12 weeks	Placebo	Reduce body weight	Bahrami et al. [27]
Menopausal women	/	5–8 mg/day for 6–12 months	Placebo	Reduce BMI	Treister-Goltzman et al. [28]
38 adults with overweight or obesity	/	3 mg/day for 12 weeks	Placebo	Reduce body weight	Mohammadi et al. [29]
Overweight women on night shift	/	3 mg/day while sleeping for 12 weeks	Placebo	Reduce body weight	Marqueze et al. [30]
Patients with cancer and cachexia	/	20 mg/day for 28 days	Placebo	No effects	Del Fabbro et al. [31]

Abbreviations: BMI: body mass index; NAFLD, non-alcoholic fatty liver disease; SGAs, second generation antipsychotics.
